# The effect of complex double-axis rotation model on GABAergic neurons in caudal ventrolateral medulla of rats along the vestibulo-sympathetic reflex pathway

**DOI:** 10.3389/fnana.2026.1749231

**Published:** 2026-04-08

**Authors:** Jian Qi, Chen Chen, Qian Gao

**Affiliations:** 1 Department of Orthopedics, The 960th Hospital of the Chinese People's Liberation Army Joint Logistic Support Force, Jinan, China; 2 Department of Pharmacy, The Second Hospital of Shandong University, Jinan, China; 3 The 960th Hospital of the Chinese People's Liberation Army Joint Logistic Support Force Postgraduate Training Base, Jinzhou Medical University, Jinan, China

**Keywords:** biotinylated dextran amine, Fluoro-Gold, immunofluorescence histochemical labeling, motion sickness, rat, the caudal raphe nuclei, the caudal ventrolateral medulla, the medial vestibular nuclei

## Abstract

**Introduction:**

Motion sickness is a common physiological disorder induced by with unusual movement exposure, characterized by conflicting motion signals that trigger vestibulo-sympathetic reflexes (VSR). These reflexes mediate autonomic responses to motion-induced stress. Previous studies have implicated the vestibular nucleus complex and the caudal ventrolateral medulla (CVLM) in VSR modulation. GABA (γ-aminobutyric acid), the primary inhibitory neurotransmitter in the central nervous system (CNS), plays a key role in both cardiovascular regulation and vestibular function. However, the specific contribution of GABAergic structures to motion sickness-related cardiovascular responses remains unclear.

**Methods:**

In this study, we combined retrograde tracing and immunofluorescence labeling to investigate GABAergic pathways using complex double-axis rotation model. Fluoro-Gold (FG) was injected into the CVLM, while biotinylated dextran amine (BDA) was delivered to the medial vestibular nuclei (MVe).

**Results:**

Our results revealed a bilateral distribution of GABAergic neurons, predominantly within the caudal raphe nuclei. Notably, a subset of these neurons was activated (as indicated by Fos immunoreactivity) and projected to the CVLM (as shown by retrograde labeling with FG) under complex double-axis rotation model. Furthermore, these same neurons also received direct inputs from the MVe, as evidenced by their labeling with BDA.

**Discussion:**

Our findings offer morphological evidence that GABAergic neurons in the caudal raphe nuclei participate in the cardiovascular responses evoked by motion sickness in a complex double-axis rotation model.

## Introduction

Motion sickness is a prevalent physiological disorder triggered by with unusual movement exposure, characterized by distressing autonomic symptoms such as pallor, nausea, vomiting, and appetite suppression, all of which impair human performance and wellbeing (Wang et al., 2012). These symptoms reflect a maladaptive response to conflicting motion signals, underscoring the vestibular system’s critical role in autonomic regulation (Gotoh et al., 2004). The vestibular system, essential for spatial orientation and balance, contributes to motion perception, and its dysfunction can precipitate motion sickness in susceptible individuals (Gotoh et al., 2004). Central to this process is the vestibular nuclear complex, the primary relay for vestibular input, which comprises four major nuclei: the medial, superior, lateral, and inferior vestibular nuclei (Khan and Chang, 2013). Notably, provocative head movements and gravitational shifts-such as those encountered in motion sickness-inducing environments-elicit significant neuroplastic adaptations within the vestibular nuclei (Abe et al., 2007; du Lac et al., 1995; Kenyon and Young, 1986).

As the largest component of the vestibular nuclear complex, the medial vestibular nucleus (MVe) forms the medial column (Khan and Chang, 2013). Dysfunction in the MVe impairs vestibulo-ocular reflexes (VOR) and is implicated in disorders such as vertigo and motion sickness (Tu et al., 2025). The vestibular system detects changes in head position and posture, initiating rapid hemodynamic adjustments to ensure stable blood pressure. These compensatory mechanisms-critical for preventing syncope during postural shifts-are orchestrated by integrative pathways that link vestibular inputs to cardiovascular control centres in the ventrolateral medulla (Holstein et al., 2014). The resulting blood flow redistribution, driven primarily by sympathetic activation, constitutes the vestibulo-sympathetic reflex (VSR) (Yates, 1992; Yates and Miller, 1994). Both the MVe and the caudal ventrolateral medulla (CVLM) have been identified as key mediators of VSR (Holstein et al., 2016), underscoring their pivotal role in motion sickness-related cardiovascular regulation.

GABA (*γ*-aminobutyric acid), the primary inhibitory neurotransmitter in the CNS, plays a critical role in cardiovascular control, as demonstrated in functional studies in rabbits (Blessing, 1990). Within the vestibular nucleus complex, numerous GABA-like immunoreactive (LI) structures have been identified (Shi et al., 2022), and GABA_A_ receptors-which mediate fast inhibitory signaling-are essential for vestibular regulation (Wang et al., 2012; Zhang et al., 2005). These findings highlight GABA’s importance in modulating vestibular processing. However, the specific contribution of GABA -LI structures to cardiovascular responses in motion sickness remains poorly understood.

Previous research has demonstrated that the GABAergic neurons in the caudal raphe nuclei play a significant role in cardiovascular regulation (Blessing, 1990). However, it remains unclear whether these caudal raphe nuclei neurons receive inputs from MVe and subsequently relay information to the CVLM in motion sickness. To investigate this pathway, we subjected animals to double-axis rotation and employed an approach combining retrograde/anterograde neural tracing with immunofluorescence techniques. Specifically, we used: (1) GAD immunostaining to identify GABAergic neuronal pathways, and (2) Fos protein labeling to map rotation-activated neurons.

## Materials and methods

### Animals and ethical considerations

All experiments were carried out on male Sprague–Dawley rats (weighing 250–300 g) obtained from laboratory animal center of the 960th Hospital of Military (Jinan, P. R. China), which were housed under controlled temperature, humidity, and a 12/12 h dark–light cycle (light on at 6:00 and off at 18:00) with standard laboratory rodent chow and water *ad libitum.* Experiment protocols were approved by the Ethics Committee for Animal Experiments of the 960th Hospital of Military (Jinan, P. R. China) (Ethical Approval No. 200067). All efforts were made to minimize the amount of suffering and number of animals used.

### Experimental groups and study design

**Figure 1:**
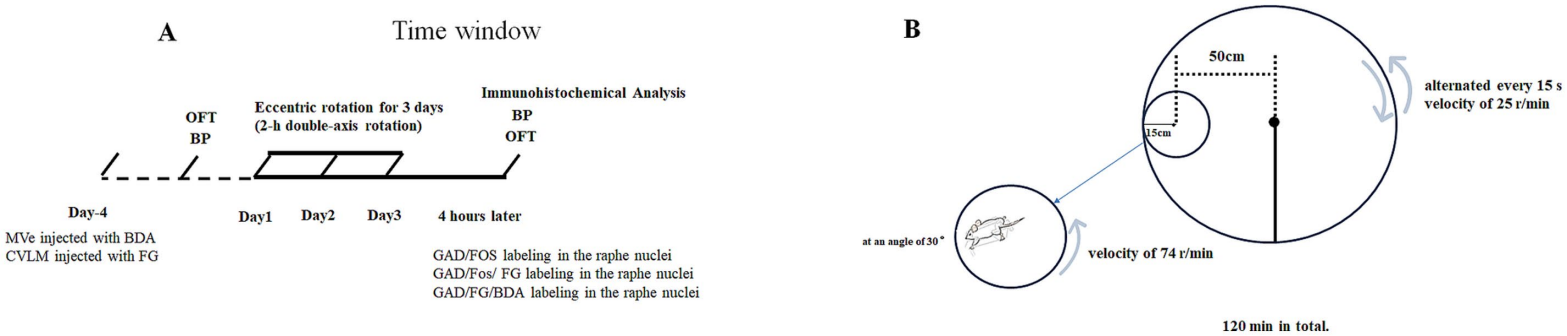
Schematic diagram of experimental design **(A)** and the graphical scheme of rotation axes illustrating centers of rotation, distances, angles and speeds of rotation **(B)**. BP, blood pressure; OFT, open field test; MVe, medial vestibular nuclei; CVLM, caudal ventrolateral medulla; BDA, biotinylated dextran amine; FG, Fluoro-Gold.

Briefly, rats were subjected to a 2-h daily protocol of complex double-axis eccentric rotation for three consecutive days (Figure 1). All animals were randomly distributed among five groups (6 animals/group) (Table 1). In groups 1 and 2, the MVe (Anterior–posterior: −6.0 mm; Medial-lateral: +0.8 mm; Dorsal-ventral: −3.2 mm) were injected with biotinylated dextran amine (BDA, anterograde tracer), while the ipsilateral CVLM (Anterior–posterior: −4.8 mm; Medial-lateral: −1.8 mm; Dorsal-ventral: −0.4 mm) received Fluoro-Gold (FG, retrograde tracer) injections. In the third group, under eccentric rotation to observe GAD/FOS labeling neurons in the caudal raphe nuclei. In the fourth group, FG was injected into the CVLM. These animals were used to examine the relationships between GAD-immunoreactive structures, Fos-positive neurons, and FG-retrogradely labeled neuronal somata in the caudal raphe nuclei under eccentric rotation. In the fifth group, BDA was injected into the MVe and FG into the ipsilateral CVLM. These animals were used to examine the relationships between MVe efferent fibers, GAD-immunoreactive structures, and FG-retrogradely labeled neuronal somata in the caudal raphe nuclei.

**Table 1:** Study cases.

Groups	*n*	Labeling	Tracer injection points
Group I	6	FG/BDA	FG administered to the CVLM and BDA to the Mve
Group II	6	FG/BDA	FG administered to the CVLM and BDA to the Mve
Group III	6	GABA/FOS	
Group IV	6	GABA/FG/FOS	FG administered to the CVLM
Group V	6	GABA/BDA/FG	FG administered to the CVLM and BDA to the Mve

### Eccentric rotation

The centrifugation apparatus was mainly composed of two horizontal disks of different size, both of which can rotate at a controlled velocity on the vertical axis situated in their centres, and the axis of the smaller disk was located 50 cm away from the axis of the larger disk. The rats for head free subjected to eccentric rotation were confined in a cage fixed on the smaller disk, 15 cm away from its axis. During the stimulation, the larger disk rotated at a constant angular velocity of 25 r/min in a clockwise direction, stop immediately, then rotated in opposite direction, so two reverse-directed rotation process alternated every 15 s and angular acceleration and deceleration were rapid. While simultaneously, the smaller disk rotated at a constant angular velocity of 74 r/min in a counterclockwise direction. The rat was oriented nose-down and head-outward at a 30° angle. The eccentric rotation went on for 120 min in total (Figure 1).

### Measurements and locomotive ability

Blood pressure was measured both before the eccentric rotation protocol and on the three consecutive days following its completion 4 h later after eccentric rotation on Day 3 (Figure 1). Animals subjected to the same procedures as the control group, with the exception of rotation. Systolic and diastolic blood pressure (SBP and DBP) were measured in conscious, unrestrained rats using a tail-cuff system (CODA Non-Invasive Blood Pressure Monitoring System, Kent Scientific). First, acclimate the rat to handling and the restrainer for 3–5 days prior to the experiment. On the measurement day, place the rat in the acrylic restrainer and position it on the heated platform (38–40 °C) for 10–15 min to dilate the tail artery. Slide the occlusion cuff onto the base of the tail, then place the VPR sensor distal to the cuff, ensuring both are snug but not constrictive. Start the automated measurement: the cuff inflates to occlude blood flow, then slowly deflates; the VPR sensor detects the return of pulse waves to determine systolic pressure and full waveform recovery to determine diastolic pressure. Record 5–10 stable cycles, discard the first 2–3 acclimation cycles, and average the remaining values to obtain systolic, diastolic, and mean arterial pressure along with heart rate. Pulse pressure (PP) was calculated as the difference between systolic and diastolic blood pressure (SBP - DBP). Mean blood pressure (MBP) was derived from the formula: MBP = DBP + PP/3.

The open field test (OFT) was used to evaluate locomotor activity after the eccentric rotation on the three consecutive days. Behavioral testing was conducted in an open-field box (1.0 m × 1.0 m × 0.4 m). After being placed in the center of the arena, animals were allowed a 3-min acclimation before their locomotion was video-recorded and analyzed for 15 min using an automated tracking system (Shanghai Mobiledatum Information Technology Co., Ltd., Shanghai China). Parameters assessed included both overall locomotor activity (total distance ambulated) and exploration of the central field (time spent and distance moved within the 0.5 m × 0.5 m central area).

### Neural tracing and immunohistochemical analysis

Animals were deeply anesthetized via intraperitoneal administration of sodium pentobarbital (50 mg/kg in 0.9% saline). Animals were positioned supine in a stereotaxic frame (Kopf Instruments, Tujunga, CA, USA) followed by surgical exposure of the medullary cisternal cavity. FG (2%, 80,014, Biotium, Hayward, CA, U.S.A) was delivered into the CVLM using 4 μA positive current pulses (7 s on/7 s off, 20 min total) via iontophoresis in 0.05 M PBS (pH 7.4). Subsequently, 0.2 μL of 10% BDA (0.2 μL of a 10% solution in 0.05 M PBS, 10,000 MW, D1956, Molecular probes, Eugene, OR, U.S.A) in 0.05 M PBS was pressure-injected into the MVe via a glass micropipette (tip diameter: 15–25 μm) connected to a 1-μL Hamilton microsyringe. After a 7 days survival period, rats were deeply anesthetized with sodium pentobarbital (40 mg/kg, i.p.) and perfused transcardially with 150 mL of 0.01 M PBS (pH 7.4), followed by 500 mL of 4% formaldehyde in 0.1 M phosphate buffer (PB, pH 7.4). Brains were post-fixed overnight in the same fixative at 4 °C, then cryoprotected in 30% sucrose/0.1 M PB until saturated (24–48 h). Brainstems were sectioned coronally at 30 μm thickness using a freezing microtome (Leitz Kryostat 1720, Mannheim, Germany). Sections were collected in 0.01 M PBS and systematically divided into five series. The first sections were examined under an epifluorescence microscope (Olympus BX-60, Japan) to map FG injection sites and retrogradely labeled neuron. The second series was processed to visualize both the BDA injection site in the MVe and the distribution of anterogradely labeled fibers throughout the brainstem. For BDA visualization, tissue sections were first permeabilized by overnight incubation in 0.5% Triton X-100/0.05 M PBS (pH 7.6) at 4 °C, followed by a 2-h room temperature incubation with Cy3-conjugated avidin D (1:200 dilution; Molecular Probes Eugene, Oregon, USA). After thorough PBS rinses, sections were mounted on gelatin-coated slides, air-dried, and coverslipped using an anti-fade mounting medium consisting of 50% glycerol and 2.5% triethylenediamine in 0.1 M PBS. BDA injection sites and their resultant fiber distributions were then systematically examined and documented using fluorescence microscopy. The third group (animals which had eccentric rotation) was used to observe GAD/Fos immunofluorescence labeling. The sections were incubated first with mouse anti-GAD67 IgG (1:500, MAB5406; Millipore, Temecula, CA, USA) and rabbit anti-Fos IgG (1:500, ab209704; Abcam, Cambridge, MA, USA) at room temperature for 12 h, and then with Alexa Fluor 488 conjugated goat anti-mouse IgG (1:400 dilution; Molecular Probes Eugene, Oregon, USA) and Alexa Fluor 594 conjugated goat anti-rabbit IgG (1:400 dilution; Molecular Probes Eugene, Oregon, USA) for 4 h. The fourth group (animals which had received exact the CVLM injections under eccentric rotation) was used to observe FG/GAD/Fos immunofluorescence labeling. Briefly, the sections were incubated first with a mixture of guinea pig anti-FG IgG, mouse anti-GAD67 IgG (1:500, MAB5406; Millipore, USA) and rabbit anti-Fos IgG (1:500, ab209704) at room temperature for 12 h, and then with Alexa Fluor 647 conjugated goat anti-rabbit IgG (1:400 dilution; Molecular Probes Eugene), Alexa Fluor 488 conjugated goat anti-mouse IgG (1:400 dilution; Molecular Probes Eugene), Cy3-labeled avidin D for 4 h. The fifth group (animals which had received exact the MVe and CVLM injections under eccentric rotation) was used to observe FG/BDA/GAD immunofluorescence labeling. Briefly, the sections were incubated first with a mixture of rabbit anti-FG IgG, mouse anti-GAD67 IgG (1:500, MAB5406) at room temperature for 12 h, and then with Alexa Fluor 647 conjugated goat anti-rabbit IgG (1:400 dilution; Molecular Probes Eugene), Alexa Fluor 488 conjugated goat anti-mouse IgG (1:400 dilution; Molecular Probes Eugene), Cy3-labeled avidin D for 4 h. The antibody incubation buffers were prepared as follows: primary antibody diluent consisted of 0.05 M PBS (pH 7.4) supplemented with 2% normal donkey serum (NDS), 0.5% Triton X-100, 0.05% sodiumazide (NaN_3_), and 0.25% *λ*-carrageenan (NDS-PBS), while secondary antibodies were diluted in 0.05 M PBS (pH 7.4) containing 0.5% Triton X-100 alone. The numbers of counted cells were corrected by use of Abercrombie’s equation: number of cell = number of cells counted × *T*/(*T* + *h*), where *T* = thickness of the sections and *h* = the mean diameter of the nuclei of the large or small cells.

### Microscopy and image analysis

All fluorescent markers-including BDA-labeled fibers, FG-labeled neurons, Fos-LI neurons, and GABAergic neurons-were examined with an Olympus Fluoview 1,000 confocal laser-scanning microscope (CLSM). The system employed three laser excitation wavelengths: 488 nm (with FITC filter, 494–518 nm emission), 543 nm (with Alexa 594 filter, 593–615 nm emission), and 640 nm (with Cy5 filter, 705 nm emission). Digital images were acquired using the manufacturer’s FLUOVIEW imaging software (Olympus, Tokyo, Japan).

## Results

### Behaviors study

**Figure 2:**
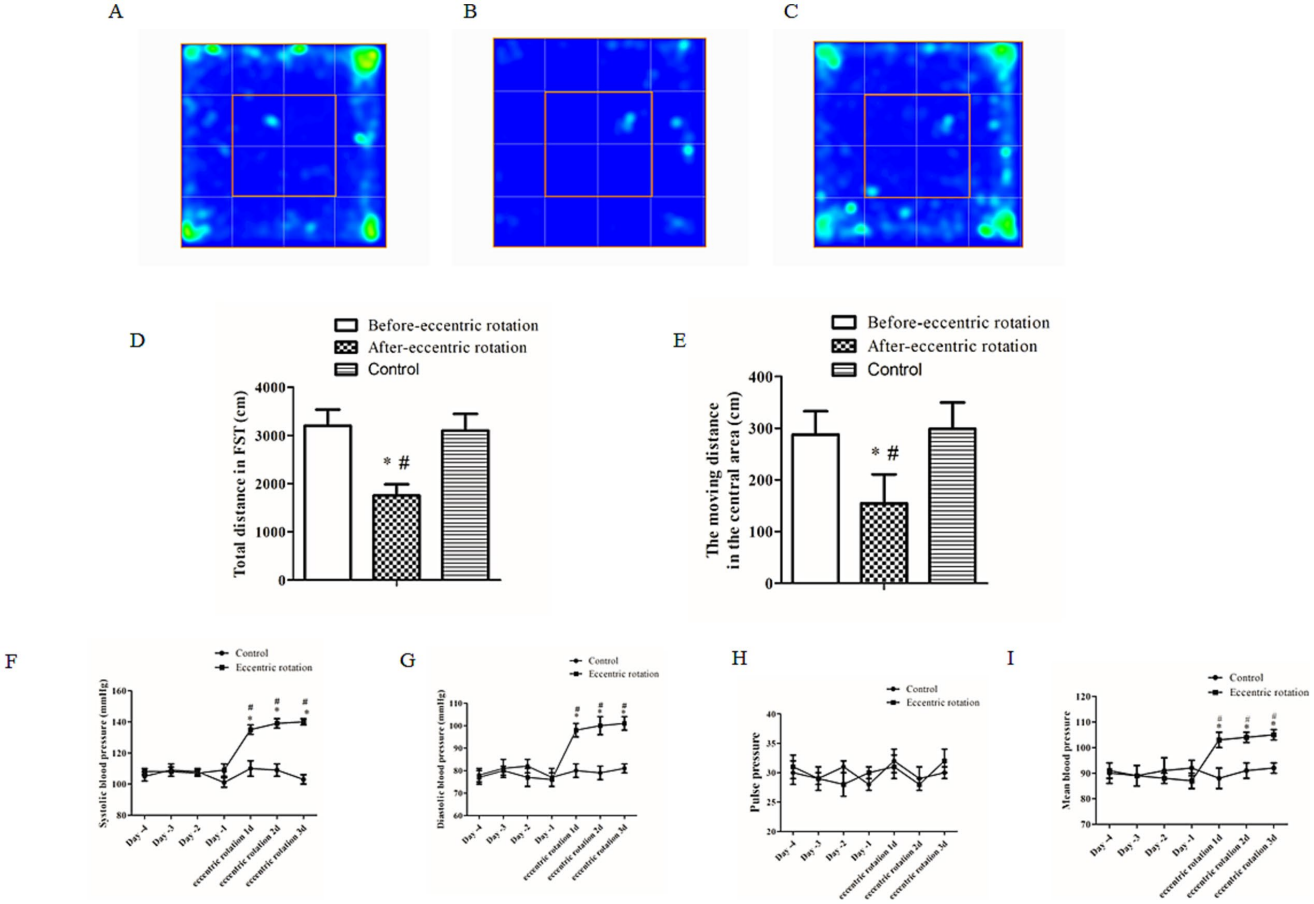
The open field test (OFT) of locomotion and blood pressure (BP) parameters. Representative OFT heat maps **(A–C)**. A comparison of the average total distance traveled **(D)** and moving distance in the central area **(E)**. Control, before and following the rotation procedure revealed a significant difference (^*^p < 0.05, *n* = 6, Wilcoxon paired-test). A comparison of the systolic pressure **(F)**, diastolic pressure **(G)**, PP **(H)** and MBP **(I)** before and following the rotation procedure revealed a significant difference (^*^p < 0.05, *n* = 6, Wilcoxon paired-test).

To determine the effect of eccentric rotation on motor function, locomotor activity was assessed using the open field test (OFT) (Figures 2A–C). The total distance traveled in the open field was significantly greater before eccentric rotation (3,204.45 ± 332.22 cm) compared to after (1,755.00 ± 234.17 cm) (*p* < 0.05, *n* = 6) (Figure 2D). The distance in the central area was significantly decrease after eccentric rotation (154.45 ± 56.23 cm) compared to before (287.76 ± 45.23) (*p* < 0.05, *n* = 6) (Figure 2E). The eccentric rotation increased systolic pressure, diastolic pressure, and MBP compared to before eccentric rotation or control group (*p* < 0.05, *n* = 6) (Figures 2F–I).

### BDA/FG immunoreactivity in the caudal raphe nuclei

**Figure 3:**
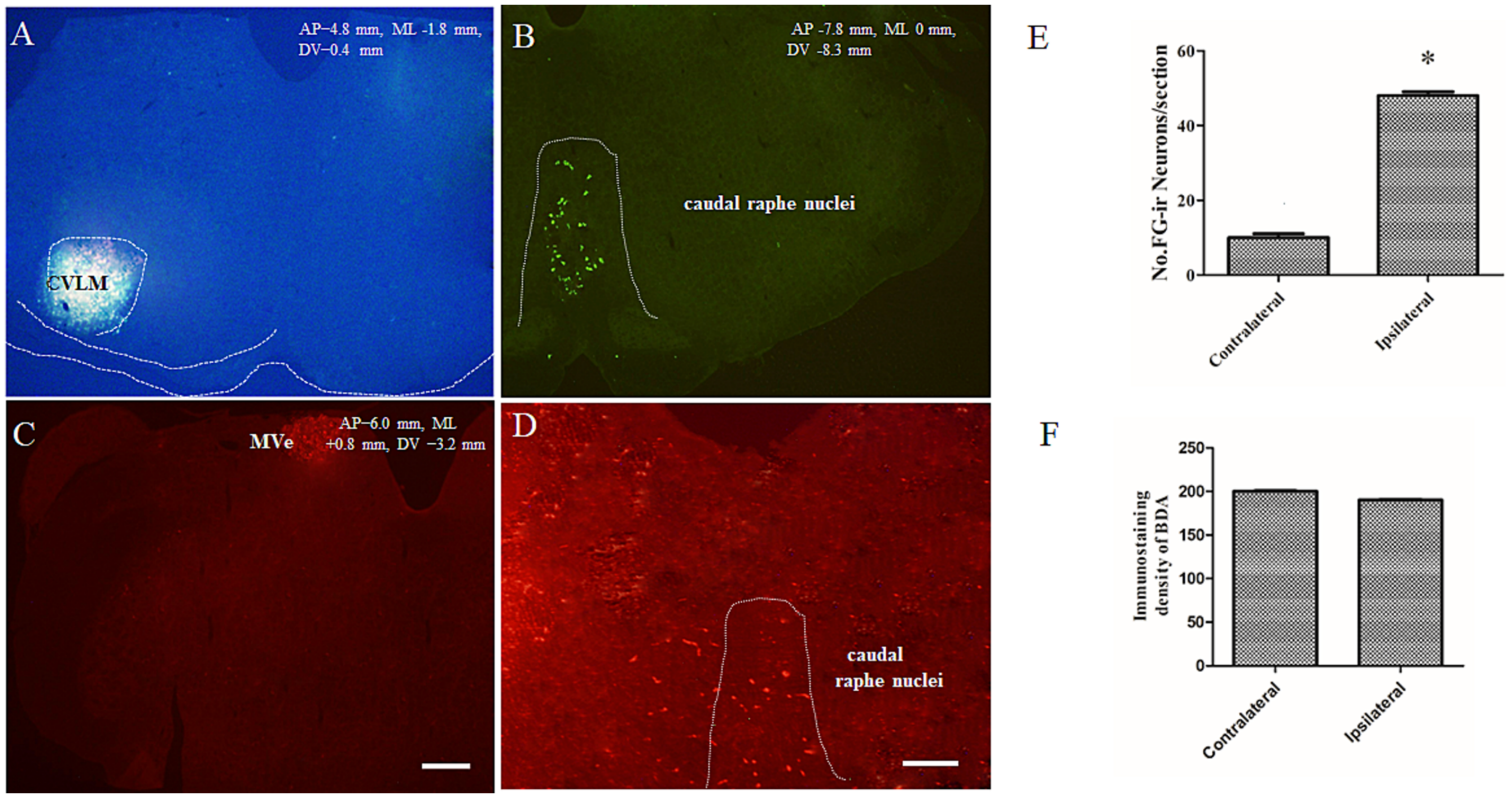
Direct appositions observed between BDA-positive terminal fibers and FG-retrogradely labeled neurons within caudal raphe nuclei. FG was injected into the CVLM **(A)**. BDA was injected into medial vestibular nuclei **(C)**. Images showing axonal terminals (red) of BDA-labeled **(D)** and FG-labeled neuronal cell body in the caudal raphe nuclei **(B)**. Scale bars = 250 μm in **(A, C)** and 25 μm in **(B, D)**. Comparison of the number of FG immunoreactive neurons in ipsilateral and contralateral within the caudal raphe nuclei **(E)**. Comparison of relative immunodensities of BDA in ipsilateral and contralateral within the caudal raphe nuclei **(F)** (^*^p < 0.05 compared to the contralateral).

**Figure 4:**
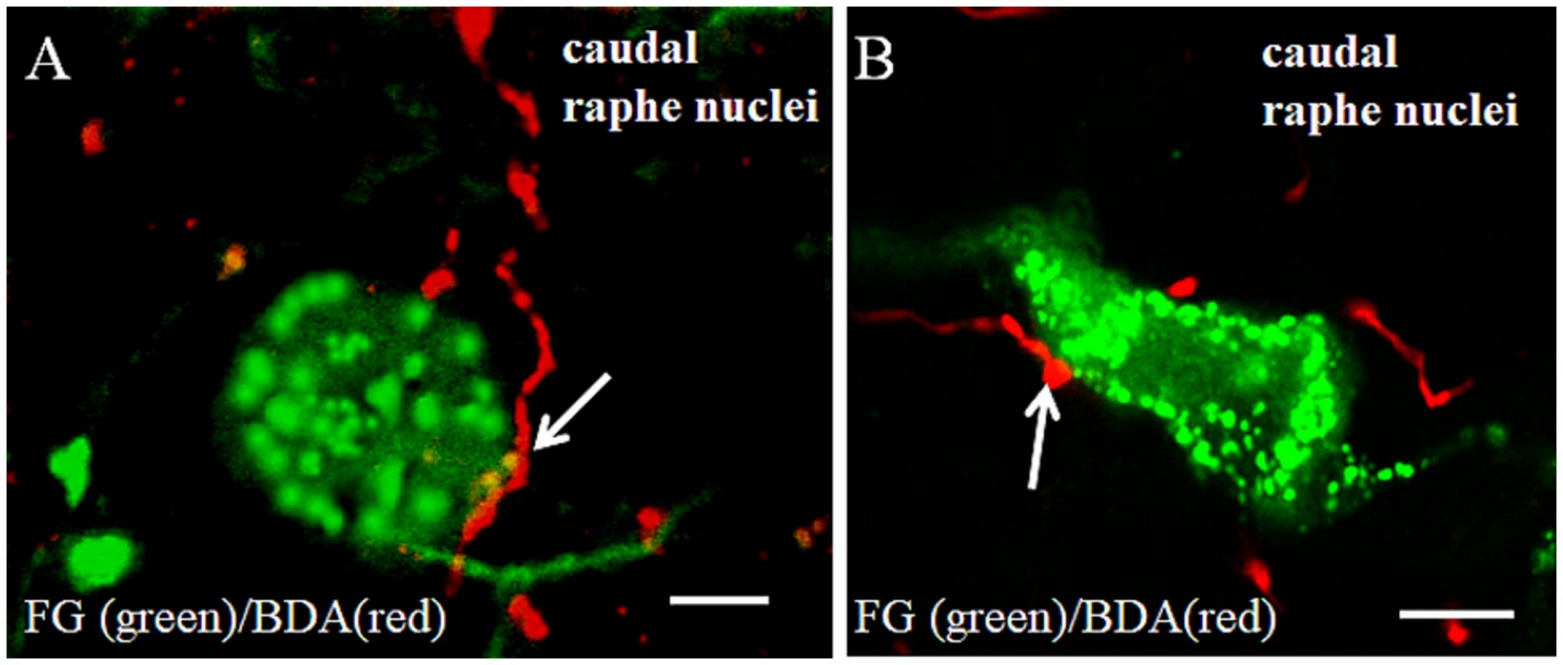
Images showing the close appositions (arrows) between axonal terminals (red) of BDA-labeled and FG (green)-labeled neuronal cell body in the caudal raphe nuclei. Scale bars = 30 μm in **(A, B)**.

Following Fluoro-Gold (FG) microinjection into the CVLM (Figure 3A), retrograde tracing revealed a predominantly ipsilateral distribution of FG-labeled neurons within the caudal raphe nuclei (*p* < 0.05) (Figures 3B,E). Subsequent biotinylated dextran amine (BDA) injection into MVe (Figure 3C) demonstrated extensive bilateral projections of BDA-labeled fibers throughout the caudal raphe nuclei (Figure 3D). There were no significant differences between the ipsilateral and the contralateral caudal raphe nuclei in relation to the fluorescence intensity of BDA (Figure 3F). High-resolution confocal imaging confirmed the presence of both FG-labeled neuronal somata (green fluorescence) and BDA-labeled axonal terminals (red fluorescence) within the same raphe regions. Immunofluorescence analysis at higher magnification revealed numerous close appositions between vestibular-derived BDA-labeled terminals and CVLM-projecting FG-labeled neurons (Figure 4), suggesting potential synaptic contacts between these neural pathways.

### Eccentric rotation-exposed rats showed significant upregulation of Fos in the caudal raphe nuclei

**Figure 5:**
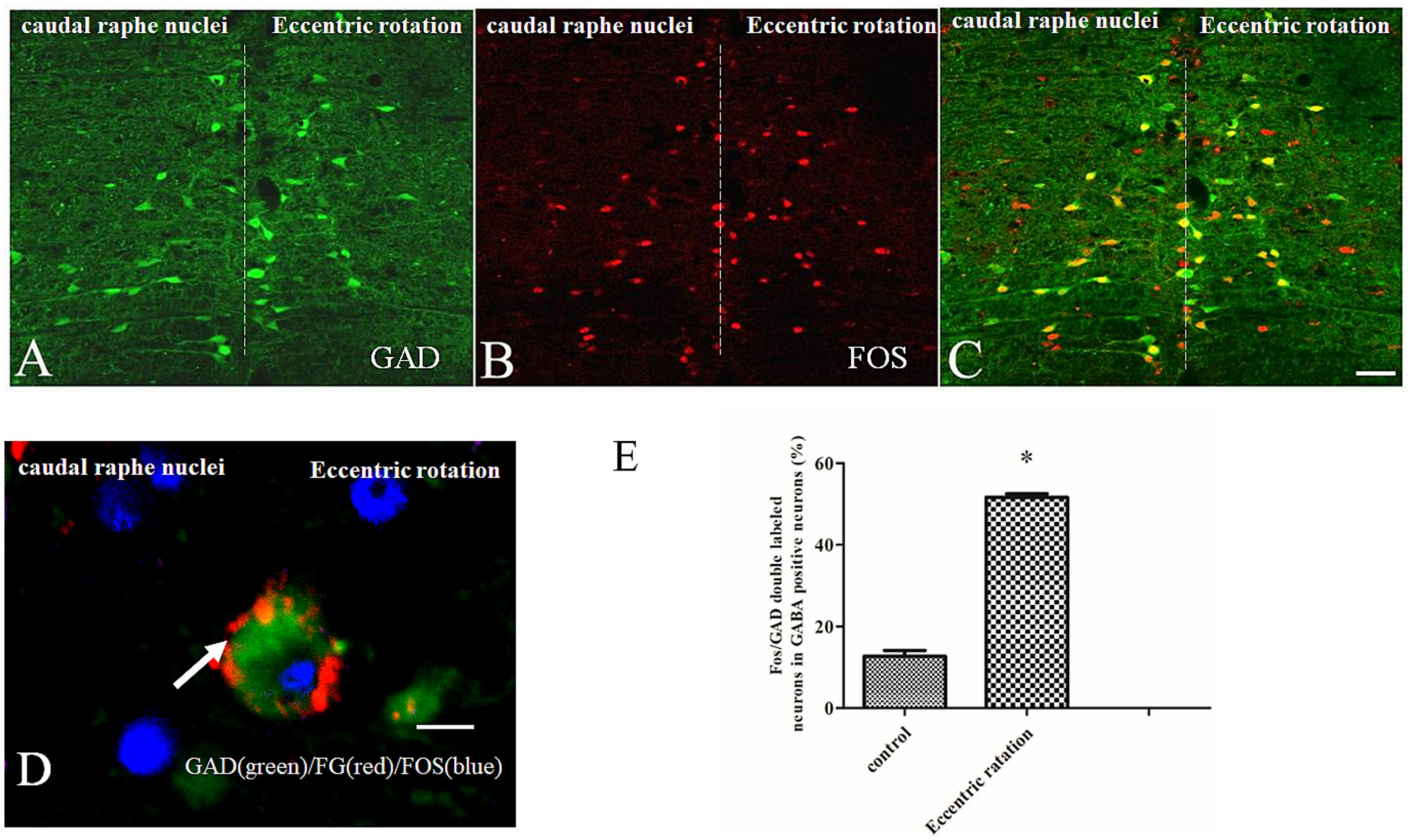
FG/Fos/GAD labeled neurons. **(A–C)** Eccentric rotation induced a significant upregulation of Fos immunoreactivity in the caudal raphe nuclei, and most Fos-positive neuron was GAD-positive. **(D)** FG was injected into the CVLM, GAD/Fos/FG-positive neurons was observed in the caudal raphe nuclei. Scale bars = 200 μm in **(A)** and 25 μm in **(B)**. **(E)** Statistical results showed No. Fos/GAD-ir neuron in GAD-positive neuron under eccentric rotation group and control group.

The caudal raphe nuclei, a cluster of neurons located along the midline of the brainstem, derive their name from their position at the junction of the left and right brainstem structures (“raphe” meaning seam or ridge). Within these nuclei, we observed GAD-immunoreactive (GAD-LI) neurons exhibiting diverse morphologies, including oval, fusiform, and irregular shapes (Figure 5A). There were significant differences between the eccentric rotation group and control group in relation to the number of Fos-ir neurons (*F*2,11 = 56.85, *p* < 0.001). The eccentric rotation model significantly increased Fos immunoreactivity compared to controls (Figures 5A,C). Notably, 65% of GAD-LI neurons were co-labeled with Fos (Figure 5A). There were significant differences between the eccentric rotation group and control group in relation to the number of Fos/GAD-double label neurons in GAD-positive cells (*F*2,11 = 54.75, *p* < 0.001). The number of Fos/GAD-double label neurons in GAD-positive cells was much more in the eccentric rotation group than that in control group (Figures 5D,E). Following FG injection into the CVLM, retrograde labeling revealed FG-positive neurons in the caudal raphe nuclei, many of which were also GAD- and Fos-immunoreactive (Figure 5B). Table 2 quantifies the proportion of FG-labeled, GAD-immunoreactive neurons in the CVLM that were co-localized with Fos.

**Table 2:** Quantification of GAD-immunoreactive neurons that were FG-labeled following CVLM injections, co-localized with Fos-positive cells.

Rat	FG-labeled neurons	Fos-LI neurons	GAD-LI neurons	FG/Fos/GAD double-labeled neurons (%^1^; %^2^, %^3^)
R1	21	54	45	10 (47.6; 18.5; 22.2)
R3	19	50	40	9 (47.3; 18; 22.5)
R5	17	51	41	8 (47.0; 15.7;19.5)
R6	22	52	42	11 (50.0; 21.1;26.2)
Total	79	207	168	38 (48.1; 18.3;22.6)

%^1^, percentage of FG/Fos/GAD double-labeled neurons to FG-labeled neurons.

%^2^, percentage of FG/Fos/GAD double-labeled neuron to Fos-IR neurons.

%^3^, percentage of FG/Fos/GAD double-labeled neuron to GAD-IR neurons.

### FG/GAD/BDA immunoreactivity in the caudal raphe nuclei

**Figure 6:**
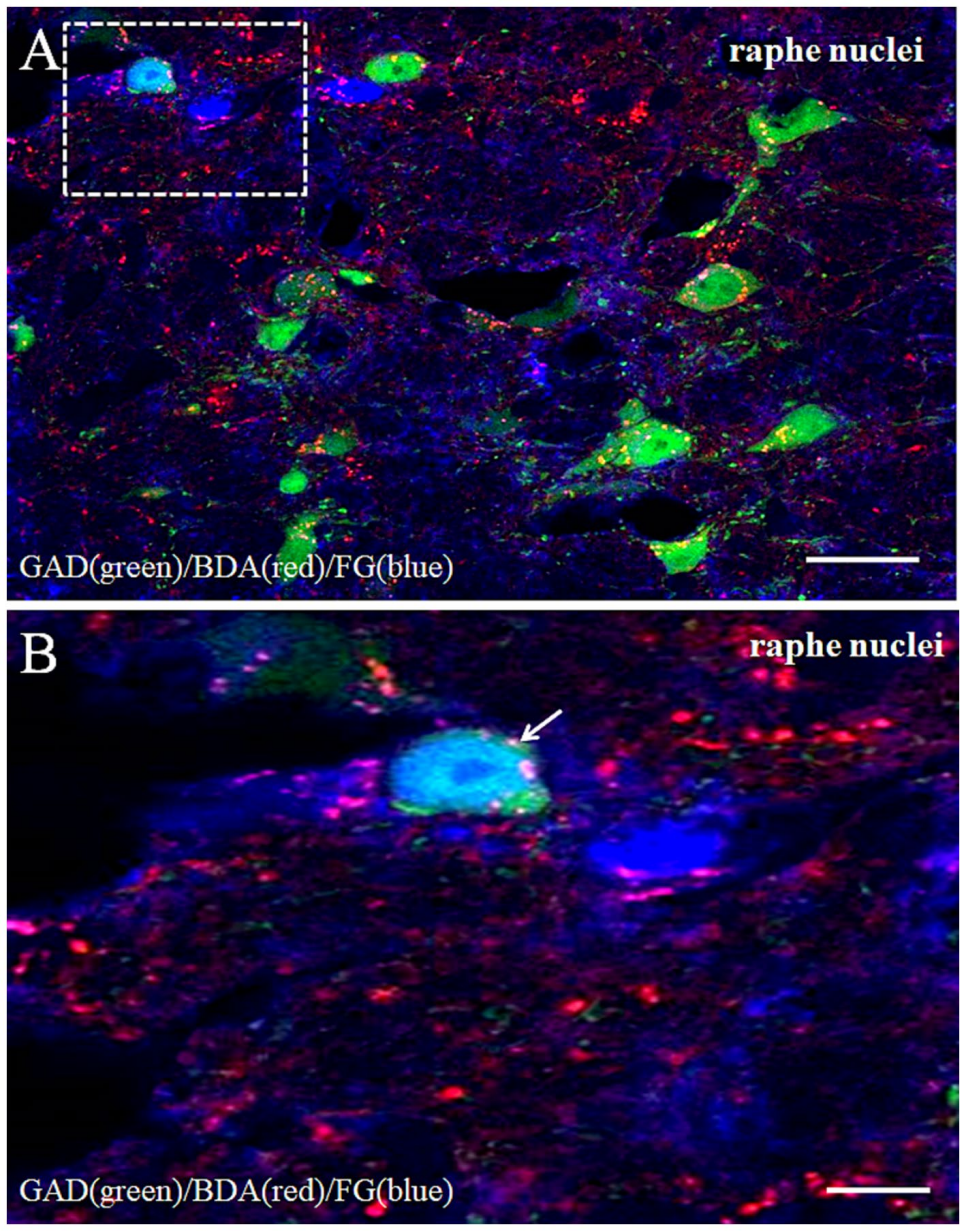
Projections showed BDA-labeled fibers from the medial vestibular nuclei making close appositions to the FG retrogradely labeled/GAD-double neuron in the caudal raphe nuclei. BDA (red, **(A)**)-labeled axonal terminals made close appositions (arrows) to the FG/GAD labeled neurons after FG injected into the CVLM and BDA into the MVe. Scale bars = 10 μm. **(B)** Are the magnified images of the rectangles represented in the panels A. Scale bars = 30 μm.

Following FG injection into the CVLM, retrogradely labeled neurons were identified in the caudal raphe nuclei. Subsequent BDA injection into the MVe revealed bilateral projections to the caudal raphe nuclei. Confocal microscopy demonstrated numerous BDA-labeled fibers and terminals forming close appositions with FG/GAD double-labeled neurons, contacting both somata and dendritic processes (Figures 6A,B).

### Statistical analysis

The number of immunoreactive neurons and fluorescence intensity of BDA were analyzed by one-way analysis of variance (ANOVA). The behavior data were analyzed by *t*-tests. The data are all expressed as means ± S.E.M. The tests were two sided. *p* < 0.05 was defined as statistical significance.

## Discussion

In this study, eccentric rotation was employed to elicit motion sickness, which subsequently triggered cardiovascular responses evidenced by changes in blood pressure. Using combined tract-tracing and immunohistochemical techniques at the light microscopic level, we investigated the neural pathways underlying the vestibulo-sympathetic reflex. Our findings demonstrate that GAD/Fos-positive neurons under eccentric rotation in the caudal raphe nuclei primarily represent FG-labeled projections from the CVLM. Combined BDA (Mve) and FG tracing with GAD immunohistochemistry revealed bilateral distribution of these inhibitory neurons in the caudal raphe nuclei.

Motion sickness represents a maladaptive physiological response to modern vehicular and aerospace environments, characterized by distressing autonomic symptoms including nausea, vomiting, pallor, and anorexia that substantially impair human performance (Wang et al., 2012). As such, investigation of motion sickness has provided crucial insights into the vestibular system’s role in autonomic regulation. Current evidence indicates the vestibular nuclear complex serves as a key mediator of autonomic adjustments during locomotion and postural alterations across mammalian species (Wang et al., 2012). Notably, experimental studies have demonstrated that both provocative cephalic movements and altered gravitational conditions induce marked neuroplastic reorganization within vestibular nuclei circuits.

The vestibular nuclear complex comprises four principal nuclei that are anatomically and functionally distinct: the medial (Schwalbe’s nucleus), superior (Bechterew’s nucleus), lateral (Deiter’s nucleus), and inferior (descending nucleus) vestibular nuclei (Khan and Chang, 2013; Dieterich and Brandt, 2001; Sakka and Vitte, 2004). These nuclei form two prominent columns beneath the floor of the fourth ventricle, extending from the caudal pons through the rostral medulla. As the largest component of the complex, the medial vestibular nucleus constitutes the medial column and serves as a primary integration center for vestibular inputs. It receives direct afferent projections from the crista ampullaris of the lateral semicircular canals, processing critical information about angular head acceleration. The medial vestibular nucleus also regulates the vestibulospinal reflex through bilateral descending projections in the medial vestibulospinal tract, which terminates in the cervical spinal cord to coordinate head and neck movements.

The vestibular system plays a critical role in cardiovascular regulation by detecting head movements and postural changes, then initiating rapid hemodynamic adjustments to preserve blood pressure homeostasis. These vital compensatory mechanisms - which prevent orthostatic hypotension and syncope during postural transitions - are mediated by specialized neural circuits that integrate vestibular information with cardiovascular control centers in the ventrolateral medulla (Holstein et al., 2014). The resulting blood flow redistribution, driven primarily by sympathetic nervous system activation, is termed the vestibulo-sympathetic reflex (VSR) (Yates et al., 2014). The medial vestibular nucleus was confirmed to have important mediatory function in the central organization of the vestibulo-sympathetic reflex (Monos and Lorant, 1998). Modulations in systemic blood pressure are monitored by baroreceptors in the carotid body and aortic arch. Second order neurons of this baroreflex pathway are located in the solitary nucleus, which sends major projections to the CVLM (Holstein et al., 2014). The CVLM, provides both excitatory and inhibitory input to the RVLM, as well as catecholaminergic projections to the hypothalamus and basal forebrain. According, cells in the CVLM have distinct and diverse roles, extending well beyond the basic circuitry of the baroreflex. Previous studies demonstrated that the vestibular nucleus complex and CVLM involves in the vestibulo-sympathetic reflex (Yates, 1992).

Gravitational head and body displacements trigger sympathetic activation, whereas linear acceleration evokes transient cardiovascular adjustments. Notably, these autonomic responses are markedly diminished in individuals with bilateral vestibular dysfunction, highlighting the essential role of vestibular inputs in cardiovascular regulation (Holstein et al., 2014). Consequently, the vestibular nucleus complex and CVLM play a critical role in regulating cardiovascular responses associated with motion sickness (Jian et al., 2005). Our results showed that BDA-labeled fibers were closely apposed to FG-labeled neurons in the caudal raphe nuclei after FG injected into the CVLM and after BDA injected into the MVe. These results indicated that the connections between the caudal raphe nuclei and CVLM or Mve. However, the neural circuitry and potential involvement of caudal raphe nuclei in motion sickness-related cardiovascular responses remain unknown. Earlier research revealed that reduced functional connectivity in the caudal raphe nuclei was associated with more pronounced motion sickness symptoms (Balaban et al., 2014). The eccentric rotation model is an experimental paradigm used to study motion sickness and vestibular-mediated autonomic responses. In this model, subjects or animals are positioned off-center on a rotating platform, creating conflicting sensory inputs between vestibular (angular acceleration) and visual/somatosensory (linear motion) systems. This mismatch induces motion sickness-like symptoms and activates vestibulo-sympathetic reflexes (VSR), mimicking physiological responses to provocative motion (e.g., spaceflight or vehicular movement). In our study, eccentric rotation was employed to elicit motion sickness, which subsequently triggered cardiovascular responses evidenced by changes in blood pressure. Fos protein, a well-established marker of neuronal activation, may serve as a molecular indicator for both motion sickness progression and habituation processes (Abe et al., 2010). In the present study, using eccentric rotation model, rats showed significant upregulation of Fos in the caudal raphe nuclei. There is some evidence that c-fos gene immunoreactivity is affected by movement and change in posture, increasing gene activity in immunoreactivity autonomic nuclei (Yuan et al., 2021). However, due to pentobarbital provides limited analgesia and suboptimal muscle relaxation, which may compromise both animal welfare and experimental outcomes Importantly, it cannot be excluded that the observed Fos immunoreactivity may, at least in part, reflect nociceptive activation due to insufficient anaesthesia. Further studies should combine such as ketamine with xylazine, with adjuncts like acepromazine, to ensure adequate analgesia, hypnosis, and muscle relaxation. These results demonstrate that the eccentric rotation model activates neurons in the caudal raphe nuclei, providing new insights that complement previous findings. Stress such as motion sickness may modulate hypothalamic–pituitary adrenal (HPA) axis through the vestibulo-paraventricular pathways. Previous studies showed that activation of HPA axis might be a transient response to provocative motion rather than a causing factor of motion-induced autonomic responses (Zhou et al., 2017).

Neurons that express GAD are present throughout the CNS and most of them are inhibitory interneurons. GABAergic neurons in various parts of the vestibular nuclear complex send projections to oculomotor neuron pools, participate in mediating commissural inhibition (Holstein et al., 2016). Functional studies in the rabbit suggest that GABA is an important inhibitory neurotransmitter in the control of cardiovascular function (Blessing, 1990) and previous results showed that GABA play an important role in modulating vestibular system information. However, the role of GABAergic structures on cardiovascular responses associated with motion sickness has not been fully investigated. In our study, after FG was injected into the CVLM, a large number of FG-labeled neurons are GAD/Fos-LI structures in the caudal raphe nuclei, and a large number of BDA-labeled fibers and terminals from Mve surrounded FG/GAD double-labeled neurons and apposed to both the soma and dendritic arborizations of these neurons using eccentric rotation model. These results provide morphological evidence that GABAergic structures in the caudal raphe nuclei have an important role in cardiovascular responses associated with motion sickness regulation in this way. This connection might enhance inhibitory control over the sympathetic nervous system. However, the functional consequences of this connection remain to be explored.

In sum, the study reveals the morphological evidence of caudal raphe nuclei GABAergic neurons closely related to cardiovascular responses associated with motion sickness in the pathway of the vestibulo-sympathetic reflex.

## Data Availability

The raw data supporting the conclusions of this article will be made available by the authors, without undue reservation.
